# Impact of 2D *h*-BN Interlayer on Leakage Mechanisms and Device Performance Optimization in High-Reliability *β*-Ga_2_O_3_ MIS Devices

**DOI:** 10.3390/nano16150961

**Published:** 2026-08-04

**Authors:** Yikun Li, Jiarui Zhang, Wenbin Liu, Lei Wang, Jinru Xie, Jintong Xu, Chenhui Yu

**Affiliations:** 1 School of Information Science and Technology, School of Microelectronics and Integrated Circuits (Jiangsu Key Laboratory of Semiconductor Device and IC Design, Package and Test), Nantong University, Nantong 226019, Chinaliuwenbin@stmail.ntu.edu.cn (W.L.); wanglei@stmail.ntu.edu.cn (L.W.); jinruxie@stmail.ntu.edu.cn (J.X.); 2 Shanghai Institute of Technical Physics, Chinese Academy of Sciences, Shanghai 200083, China

**Keywords:** *β*-Ga_2_O_3_, *h*-BN, Fowler–Nordheim tunneling, TCAD, SRH recombination, Ga_2_O_3_ based MIS

## Abstract

The ultra-wide bandgap semiconductor *β*-Ga_2_O_3_ is a promising material for next-generation optoelectronic systems and hybrid nanodevices. However, high interface state densities and anomalous trap-assisted leakage severely restrict its performance and signal transduction capabilities. To resolve these fundamental limitations, we investigated a two-dimensional *h*-BN interlayer to construct a high-quality heterogeneous metal/*h*-BN/*β*-Ga_2_O_3_ structure using experimentally calibrated Sentaurus TCAD simulations. Energy-band analysis and validated *I*–*V* simulations reveal that the low-dimensional *h*-BN interlayer reconstructs the interfacial barrier, suppresses interface-assisted recombination, and shifts the dominant carrier transport from thermionic emission to Fowler–Nordheim tunneling. These effects markedly reduce the interface-state density and effectively suppress the Shockley–Read–Hall recombination current, mechanisms that are critical for minimizing dark current and improving device sensitivity. After systematically examining the effects of key parameters on the electrical characteristics of this hybrid architecture, we quantify the tradeoff between threshold voltage and on-resistance using a comprehensive figure of merit. Specifically, our results indicate that maximum device efficiency is achieved only when an optimal *h*-BN thickness of 3.56–5.88 nm (10–17 atomic layers) is strategically integrated with the appropriate metal work function and semiconductor doping. Overall, this work suggests the potential advantage of 2D *h*-BN in mitigating the interfacial bottleneck of traditional *β*-Ga_2_O_3_ platforms, providing quantitative design guidelines and theoretical support for the heterogeneous integration of next-generation optoelectronic devices.

## 1. Introduction

The rapid evolution of high-power and high-frequency electronics has driven the transition from silicon-based devices to wide bandgap and ultra-wide bandgap semiconductors [1,2,3]. Among these, *β*-Ga_2_O_3_ has emerged as a leading candidate due to its demonstrated device potential and favorable intrinsic material properties [4,5]. In particular, *β*-Ga_2_O_3_ possesses an exceptional bandgap of 4.85 eV and a theoretical critical breakdown field of 8 MV/cm, which significantly surpasses that of SiC and GaN [1,5,6,7]. These intrinsic properties suggest that *β*-Ga_2_O_3_ can deliver a significantly superior Baliga figure of merit (BFOM), with a theoretical value of around 3000, enabling power devices with reduced on-resistance (*R*_on_) at a given breakdown voltage [7]. Beyond power switching capabilities, the ultra-wide bandgap of *β*-Ga_2_O_3_ inherently establishes it as a premier material for next-generation optoelectronic systems, most notably in solar blind deep-ultraviolet photodetectors where minimizing dark current and interface states is essential for achieving high sensitivity and detectivity [8,9]. Indeed, recent highly innovative research has demonstrated that Ga_2_O_3_-based solar-blind photodetectors hold immense potential across a wide range of critical fields, spanning from civilian environmental monitoring to cutting-edge military applications such as missile detection, highlighting the critical need for advanced device engineering to meet the stringent performance demands of these high-stakes scenarios [10]. Furthermore, the extraordinary properties of Ga_2_O_3_ are simultaneously driving emerging frontiers in advanced luminescent devices, as evidenced by recent breakthroughs in thermally stable europium-doped ceramics [11]. However, realizing this potential requires overcoming significant challenges in device architecture innovation and process optimization, similar to the hurdles faced in the scaling of other advanced semiconductor devices [12].

A critical bottleneck in *β*-Ga_2_O_3_ device performance is the quality of the metal–semiconductor (MS) interface. Conventional Schottky contacts often suffer from high densities of interface/surface traps together with barrier inhomogeneity, which lead to excessive leakage current, severe hysteresis, and premature breakdown [13,14,15,16]. The deleterious effect of these interface traps on dark current and overall device stability is a universal hurdle in advanced heterostructure engineering [17]. Recent advances in III-nitride heterostructure engineering further highlight the critical role of heterointerface design in regulating carrier transport and device functionality. Bias-controlled AlScN/GaN photodetectors exhibit switchable detection and synaptic modes, while optically tunable AlGaN/GaN transistors achieve low dark current and adjustable synaptic plasticity [18,19]. To validate the modeling baseline, reference metal/*β*-Ga_2_O_3_ Schottky diodes were calibrated using experimental *I*–*V* data. Specifically, reproducing the anomalous low-voltage excess current required incorporating a deep-level bulk trap in *β*-Ga_2_O_3_ to enable trap-assisted tunneling (TAT). This rigorous calibration simultaneously verifies the intrinsic *β*-Ga_2_O_3_ mobility parameters and highlights the nonideal, defect-driven leakage pathways in conventional MS contacts, calling for an advanced interfacial passivation strategy.

To fulfill this requirement, integrating two-dimensional (2D) layered materials, particularly hexagonal boron nitride (*h*-BN), provides a robust van der Waals passivation strategy to mitigate surface instabilities [20,21,22]. Given that dangling bond energetics fundamentally govern the formation of interfacial defect states in nanostructured systems [23], the atomically flat and dangling-bond-free lattice of *h*-BN enables the formation of pristine vdW heterostructures, thereby ensuring highly effective surface passivation [20,21,24]. As bandgap engineering and interface control have been pivotal in tuning intersubband transitions in AlGaN/GaN quantum wells [25], the precise engineering of the *h*-BN/*β*-Ga_2_O_3_ interface offers a pathway to tune carrier transport mechanisms [20,22]. Specifically, inserting an ultrathin *h*-BN insulating layer can promote tunneling dominated transport, suppress leakage, and maintain efficient on-state conduction [20,22,26,27,28]. Recent MOCVD studies have demonstrated wafer-scale few-layer *h*-BN films with excellent thickness uniformity and subnanometer surface roughness, indicating progress toward scalable and controllable integration [29,30].

Advanced theoretical modeling significantly accelerates the integration of 2D materials into wide-bandgap semiconductors. Notably, ab initio simulations and molecular dynamics have successfully guided the experimental realization of 2D layers at complex interfaces, including buckled 2D GaN and ultrathin Ga_2_O_3_ [31]. Inspired by such computationally driven paradigms, our study utilizes multidimensional physical simulations to rigorously evaluate the *h*-BN/*β*-Ga_2_O_3_ heterointerface.

To establish a reliable baseline for the *h*-BN passivation, we independently calibrated the tunneling characteristics of the dielectric using an experimental metal/*h*-BN/metal MIM structure. Isolating the *h*-BN layer from semiconductor band-bending effects allowed us to precisely extract the fundamental Fowler–Nordheim (F-N) tunneling parameters, including the empirical electron effective mass and barrier properties.

Built upon these MS and MIM foundational models, this work presents a comprehensive TCAD simulation study of metal/*h*-BN/*β*-Ga_2_O_3_ MIS structures. We systematically analyze the impact of the *h*-BN interlayer on device performance metrics and investigate the physical mechanisms governing vertical carrier transport. A parametric study is conducted considering five primary variables: the number of *h*-BN atomic layers, the metal work function (WF), the interface trap density (*D*_it_), the n-type *β*-Ga_2_O_3_ doping concentration, and the inclusion of Shockley–Read–Hall (SRH) recombination model. We evaluate the dependence of key performance indicators on these variables, including the threshold voltage (*V*_th_), *R*_on_, on-current (*I*_on_), and the resultant power device figure of merit (FOM). Our results demonstrate that optimizing the *h*-BN interlayer thickness and interface quality significantly enhances the FOM, thereby validating the potential of *h*-BN passivation for next-generation *β*-Ga_2_O_3_ power electronics and optoelectronic applications.

Although grounded in rigorously calibrated simulations, these theoretical models cannot fully account for highly complex fabrication realities, such as process-induced interfacial defects and 2D material transfer variations. Therefore, the primary objective of this work is to establish a rigorous predictive framework that narrows the vast structural design space, thereby providing critical quantitative guidelines for future experimental integration.

## 2. Physical Parameters and Models

To comprehensively evaluate the internal physical mechanisms and macroscopic electrical behavior of the proposed MIS diodes, we performed advanced numerical simulations using the Sentaurus TCAD. Specifically, the simulation framework develops accurate models of both the reference Metal/*β*-Ga_2_O_3_ Schottky junction and the Metal/*h*-BN/*β*-Ga_2_O_3_ heterostructure. Furthermore, the TCAD solver self-consistently computes Poisson’s equation and the carrier continuity equations, which effectively elucidates the complex interfacial transport and tunneling behaviors with physical rigor [32].

**Figure 1:**
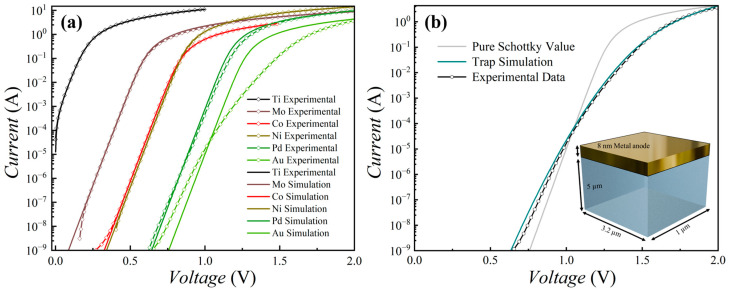
(**a**) Sentaurus TCAD calibration of the baseline metal/*β*-Ga_2_O_3_ Schottky barrier diodes. (**a**) Experimental (symbols) and simulated (solid lines) forward *I*–*V* characteristics for various anode metals. (**b**) Comparison of the experimental forward *I*–*V* characteristics for the Au/*β*-Ga_2_O_3_ contact with the pure Schottky model and the simulation incorporating a discrete acceptor trap.

As shown in Figure 1a, the simulation framework for the baseline metal/*β*-Ga_2_O_3_ MS structure was first calibrated to match the experimental measurements [15]. Free from the complexities of interfacial tunneling, this MS calibration directly validates the accuracy of the adopted mobility model in reproducing the intrinsic conduction behavior of *β*-Ga_2_O_3_, establishing a reliable baseline for the subsequent MIS analysis [32,33,34]. When each calibrated parameter was varied independently by ±5%, only minor changes were observed in the simulated *I*–*V* characteristics, indicating limited sensitivity to small parameter variations.

The key physical parameters implemented in the simulation framework are summarized in Table 1. We employed the Arora mobility model to describe carrier transport in *β*-Ga_2_O_3_, which reproduces the experimental electron mobility trend over the doping range of 10^15^ to 10^19^ cm^−3^ [32,33,34]. Therefore, the model consistently describes mobility degradation across contact regions with different doping levels [32]. Furthermore, carrier generation and recombination were modeled by three mechanisms: SRH, Auger, and radiative recombination. The SRH recombination rates are expressed as [35]:

(1) $$R_{net}^{SRH}=\frac{np-n_{i}^{2}}{\tau_{p}\left(n+n_{i}exp\left(\frac{E_{trap}}{kT}\right)\right)+\tau_{n}\left(p+n_{i}exp\left(\frac{{-E}_{trap}}{kT}\right)\right)}$$

While the simulated forward *I*–*V* characteristics of the Ti, Mo, Co, Ni, and Pd contacts agree well with the experimental data, the Au/*β*-Ga_2_O_3_ contact exhibits an unusually large ideality factor and a pronounced excess current at low forward bias. Rather than following a single exponential dependence, the substantial low-voltage leakage suggests the involvement of additional transport channels across the junction. To reproduce this anomalous behavior and elucidate the underlying transport mechanisms, we incorporated a discrete deep-level bulk trap into the TCAD model. As shown in Figure 1b, the simulated *I*–*V* curve agrees well with the experimental data when a near-midgap trap state at *E*_C_—2.40 eV is incorporated, with a concentration of 1.04 × 10^15^ cm^−3^ and a capture cross section of 10^−13^ cm^2^. The resulting simulation agrees well with the measured *I–V* characteristics [15]. In the model, this trap facilitates trap-assisted tunneling and defect-mediated hopping. Accordingly, the fitted trap parameters provide an effective characterization of the dominant defect-assisted transport process in the model.

This anomalous transport behavior may be associated with interfacial inhomogeneity at the Au/*β*-Ga_2_O_3_ contact. Previous studies have shown that defects and surface states can strongly affect the electrical characteristics of *β*-Ga_2_O_3_ Schottky contacts [36], while the large ideality factor and multi-slope *I*–*V* characteristics of Au contacts have been associated with spatially inhomogeneous Schottky barriers [15]. Moreover, nanoscale STEM and EDS analyses have revealed interfacial chemical reactions, Ga diffusion into the Au layer, and subsurface nanovoids at the Au/*β*-Ga_2_O_3_ interface [15,37]. Distributed interface states may also provide additional carrier-transport pathways at low forward bias [38]. Therefore, the excess current may arise from the combined effects of barrier inhomogeneity, interfacial reactions, and interface states, leading to an enlarged ideality factor and multi-slope semilogarithmic *I*–*V* behavior.

**Table 1:** Fundamental physical parameters for *β*-Ga_2_O_3_ material used in this simulation.

Physical 2.	Models	Parameters	*β*-Ga_2_O_3_
BandGap		Reference Bandgap (E_g0_) [eV]	4.85 eV [39]
		Reference Electron Affinity (Chi_0_) [eV]	4.0 eV [39]
		Alpha	4.45 × 10^−3^ eV/k [32]
		Beta	2000 K [32]
		Chi0	3.6128 eV [32]
Mobility	Arora Model	A_min_	13; 0.016 [40]
		α_m_	−0.57; −0.57 [40]
		A_d_	235; 0.2 [40]
		α_d_	0.78; 0.78 [40]
		A_N_	1.1 × 10^18^; 1.25 × 10^17^ [40]
		α_N_	2.4; 2.4 [40]
		A_a_	0.78; 0.78 [40]
		α_a_	−0.146; −0.146 [40]
		A_min_	13; 0.016 [40]
Recombination	SRH Recombination	Electron Lifetime (τ*_n_*); Hole Lifetime (τ*_p_*) [s]	0.2 ns; 21 ns [41]
Other basic Parameters		Dielectric Constant	10 [39]
		Effective Electron mass [*m*_0_]	0.28 [39]
		Effective Hole mass [*m*_0_]	3.4 [42]
		Effective Conduction Band Density of states N_c_ [cm^−3^]	3.72 × 10^18^ [32]
		Effective Valence Band Density of states N_v_ [cm^−3^]	3.72 × 10^18^ [43]

To isolate and calibrate the electrical properties of the *h*-BN dielectric, we next investigated a MIM structure. In the absence of semiconductor-induced band bending and carrier dynamics, this structure enables independent calibration of the *h*-BN transport model for the subsequent MIS simulations.

In the relevant high-field regime, carrier transport through *h*-BN is governed by F–N tunneling, and the theoretical expression is expressed as [44]:

(2) $$J_{F-N}=AF_{ins}^{2}\exp\left(-\frac{B}{F_{ins}}\right)$$

(3) $$A=\frac{q^{3}m}{8\pi \varphi_{B}m^{*}}$$

(4) $$B=\frac{8\pi \sqrt{2m^{*}}\varphi_{B}^{\frac{3}{2}}}{3hq}$$

**Figure 2:**
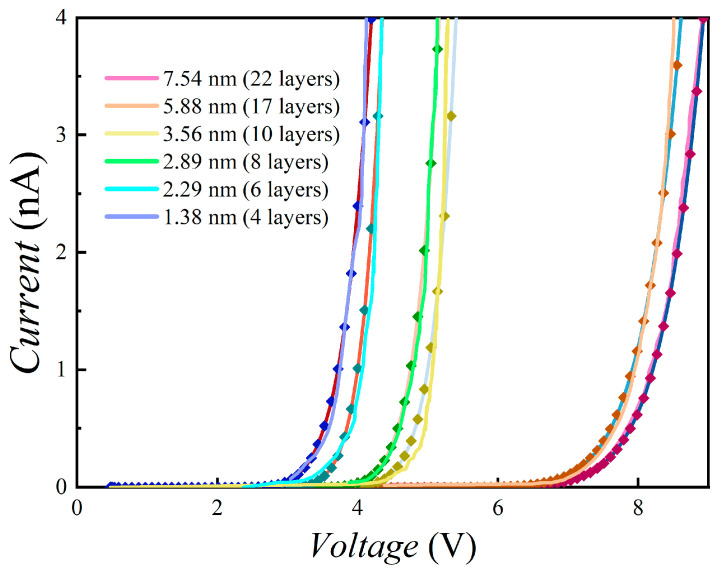
Experimental (symbols) and simulated (solid lines) *I*–*V* characteristics of metal/*h*-BN/metal structures for the extraction of F-N tunneling parameters.

According to the model, the tunneling current is exponentially sensitive to the intrinsic properties of the dielectric, the electron effective mass (*m*^∗^) and the effective interfacial barrier height (*φ_B_*). To accurately capture this quantum transport behavior, we executed a rigorous comparative parameter fitting procedure. As illustrated in Figure 2, the simulated F–N tunneling currents were systematically calibrated against experimental *I*–*V* characteristics across a comprehensive range of *h*-BN thicknesses, spanning from 1.38 nm (4 layers) up to 7.54 nm (22 layers). The exceptional agreement between the numerical model (solid lines) and the experimental data (symbols) across all thickness variants confirms the physical validity of our extraction. Through this multi-thickness global fitting, we quantitatively determined the optimal *m*^∗^ and *φ_B_*, thereby uniquely defining the fundamental tunneling parameters *A* and *B*. These calculated values, alongside other key extracted parameters, are summarized in Table 2. This meticulous multi-thickness calibration robustly establishes the intrinsic physical baseline of the *h*-BN dielectric, providing a highly reliable modeling foundation for the subsequent MIS simulations.

## 3. Results and Discussion

**Figure 3:**
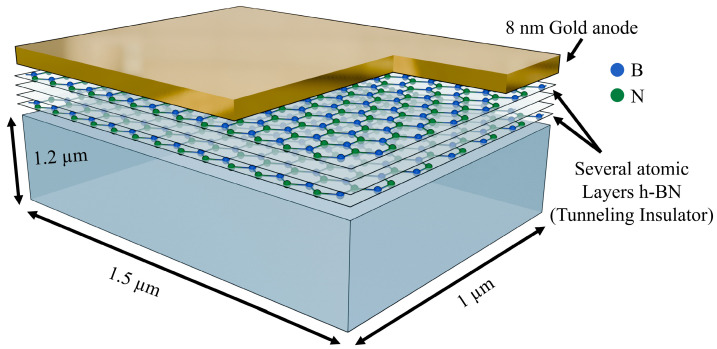
3D schematic of the proposed Au/*h*-BN/*β*-Ga_2_O_3_ MIS device. The structure consists of an 8 nm thick Au anode, an ultrathin *h*-BN tunneling insulator (several atomic layers), and a 1.2 μm thick n-type *β*-Ga_2_O_3_ base with rectangular dimensions of 1.5 μm × 1 μm.

Figure 3 illustrates the 3D schematic of the vertical Au/*h*-BN/n-type *β*-Ga_2_O_3_ MIS device. Specifically, an ultrathin *h*-BN tunneling insulator is integrated onto a 1.2 μm thick n-type *β*-Ga_2_O_3_ substrate, followed by the deposition of an 8 nm thick gold anode. The lateral dimensions of the device are fixed at 1.5 μm × 1 μm. Within this MIS heterostructure, the ultrathin *h*-BN layer effectively modulates the charge transport characteristics at the MS interface by providing an atomically sharp, high-quality van der Waals heterointerface [20,22].

**Figure 4:**
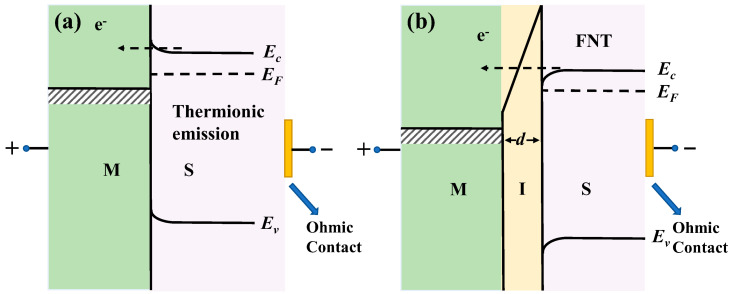
Energy-band diagrams under a large positive bias. (**a**) Metal/n-type β-Ga_2_O_3_ MS contact with thermionic emission. (**b**) MIS contact with an *h*-BN tunneling barrier and F–N tunneling. The black dashed arrows indicate electron transport, the blue arrows indicate the ohmic contacts, and *d* denotes the *h*-BN thickness.

To understand how this van der Waals configuration influences the electrical behavior of the device, we first analyze the evolution of the interfacial energy bands and the associated carrier transport mechanisms. Although the MS and MIS devices exhibit similar forward rectification characteristics, the insertion of *h*-BN modifies the dominant carrier injection process. As shown in Figure 4, electron transport across the Schottky barrier in the conventional MS structure is primarily governed by thermionic emission. By contrast, the atomically thin *h*-BN layer introduces an additional tunneling barrier in the MIS architecture, where a large forward bias is distributed between the ultrathin dielectric layer and the semiconductor depletion region. The resulting voltage drop across *h*-BN strongly tilts its energy bands and forms a triangular-like potential barrier, while simultaneously modulating the band bending in the semiconductor. Consequently, the intense localized electric field substantially narrows the effective tunneling width and enhances the contribution of Fowler–Nordheim field-assisted tunneling to carrier injection [45]. Therefore, the insertion of the *h*-BN layer reshapes the interfacial energy-band configuration, converting the single Schottky barrier of the conventional MS contact into a composite barrier system of field-assisted tunneling.

**Figure 5:**
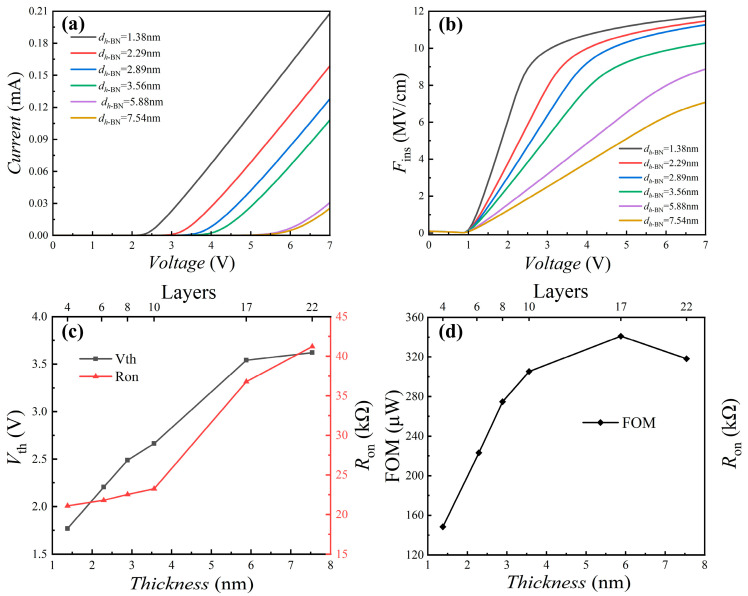
Electrical characteristics of the MIS structure (WF = 5.1 eV, doping = 1 × 10^16^ cm^−3^): (**a**) Forward *I*–*V* characteristics for different *h*-BN thicknesses (*d_h_*_-BN_ = 1.38, 2.29, 2.89, 3.56, 5.88, and 7.54 nm). (**b**) Electric field magnitude in the *h*-BN layer as a function of applied voltage for different *h*-BN thicknesses. (**c**) *V*_th_ and *R*_on_ of the MIS structure at high bias as functions of *h*-BN thickness. (**d**) FOM as a function of *h*-BN thickness.

Under the composite barrier, the forward current is primarily limited by F-N tunneling. As illustrated in Figure 5a, increasing *d_h_*_-BN_ from 1.38 to 7.54 nm exponentially suppresses the forward current. Concurrently, the device exhibits a pronounced positive shift in its turn-on characteristics, which is manifested by a monotonic increase in both *V*_th_ (the point where the measured current equals 1 × 10^−11^ A) and *R*_on_ (extracting the gradient from the highly linear portion of the current–voltage profile). This behavior can be elucidated by the steady-state internal electrostatics shown in Figure 5b. Due to the large dielectric mismatch, the applied forward bias predominantly drops across the highly resistive *h*-BN layer [46]. This concentrated voltage drop strongly tilts the insulator energy bands and redistributes the internal electric field, locally amplifying *F*_ins_ to the critical level required to trigger F-N tunneling [47]. However, under a constant external bias, a thicker *h*-BN layer inherently attenuates this internal field [48]. Therefore, inducing sufficient band tilting to sustain the critical tunneling field necessitates a higher external voltage, thereby directly accounting for the increased *V*_th_.

The extracted static parameters in Figure 5c further corroborate these electrostatic constraints. While a thicker tunneling layer enhances *V*_th_, it simultaneously introduces a larger equivalent series resistance, driving up *R*_on_. To comprehensively evaluate this intrinsic trade-off between voltage-blocking capability and forward-conduction performance, a power figure of merit, defined as FOM = *V*_th_^2^/*R*_on_, is introduced [49]. As shown in Figure 5d, the FOM exhibits a distinct nonmonotonic dependence on *d_h_*_-BN_. For *d_h_*_-BN_ ≤ 5.88 nm, the FOM increases because the quadratic enhancement in *V*_th_ outpaces the rise in *R*_on_. However, once the thickness exceeds the optimal value of 5.88 nm (17 layers), the severe degradation in forward conduction (surge in *R*_on_) becomes the dominant factor, leading to a rapid decline in the FOM. These results underscore that precise control of the *h*-BN thickness is imperative for optimizing the overall performance of *β*-Ga_2_O_3_ MIS power devices.

**Figure 6:**
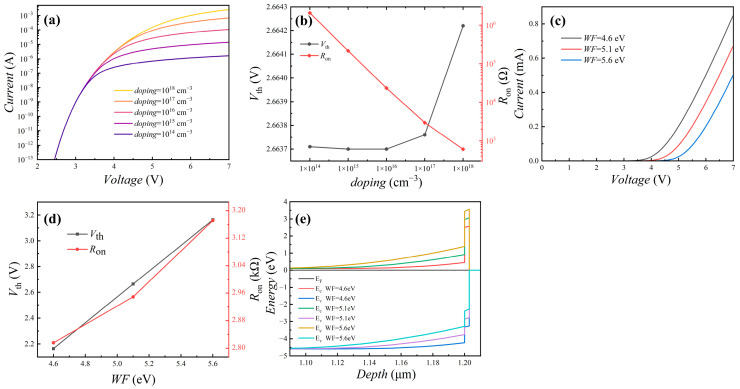
Electrical characteristics of the MIS structure as functions of *β*-Ga_2_O_3_ doping concentration and metal WF. (**a**) Forward *I*–*V* characteristics at different doping concentrations ranging from 10^14^–10^18^ cm^−3^. (**b**) Extracted *V*_th_ and *R*_on_ versus doping concentration. (**c**) Forward *I*–*V* characteristics under different metal work functions. (*d_h_*_-BN_ = 2.89 nm, WF = 5.1 eV) (**d**) Extracted *V*_th_ and *R*_on_ versus metal WF. (**e**) Zero-bias energy-band structures under different metal work functions (*d_h_*_-BN_ = 2.89 nm, doping = 1 × 10^17^ cm^−3^).

To clarify the respective roles of the *β*-Ga_2_O_3_ drift region and the metal/*h*-BN interface in forward conduction, the effects of the *β*-Ga_2_O_3_ doping concentration and metal WF are examined in Figure 6a,b. An increasing doping has a negligible influence on the low-bias characteristics, with the *I*–*V* curves nearly overlapping below 3.3 V and *V*_th_ varying by only about 0.55 mV. At higher forward biases, however, a higher doping concentration produces a larger forward current and a lower *R*_on_. This behavior indicates that the low-bias turn-on is primarily governed by F–N tunneling through the *h*-BN layer, whereas the series resistance of the drift region dominates the conduction at higher biases. In contrast, the metal WF directly modulates the interfacial potential barrier [50]. As illustrated by the energy-band diagram in Figure 6e, a higher WF enhances the band bending in *β*-Ga_2_O_3_, thereby increasing the effective electron transport barrier at the metal/*h*-BN/*β*-Ga_2_O_3_ junction. Consequently, the forward *I*–*V* curves in Figure 6c shift toward higher voltages with increasing metal WF, accompanied by a monotonic rise in both *V*_th_ and *R*_on_, as shown in Figure 6d. These results demonstrate that the bulk doping of *β*-Ga_2_O_3_ primarily determines the high-bias conduction resistance, whereas the WF fundamentally governs the interfacial energetics and the turn-on characteristics.

To evaluate the influence of the *h*-BN/*β*-Ga_2_O_3_ interface quality on device performance, this work systematically investigated the effects of *D*_it_, SRH recombination on the electrical characteristics. Conventional three-dimensional oxide/*β*-Ga_2_O_3_ interfaces, such as ALD SiO_2_ or HfO_2_, typically suffer from a high density of interface states, with a baseline *D*_it_ commonly on the order of 6 × 10^11^ cm^−2^ eV^−1^ [51], together with complex polar and border traps [52]. In contrast, *h*-BN provides an atomically flat surface without dangling bonds, thereby enabling the formation of a near-ideal van der Waals heterointerface with substantially reduced defect density.

**Figure 7:**
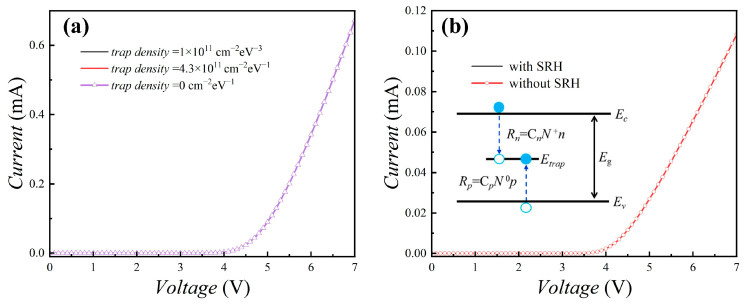
Forward conduction characteristics of the MIS structure under different interface and recombination conditions. (WF = 5.1 eV, *d_h_*_-BN_ = 2.89 nm, doping = 1 × 10^17^ cm^−3^) (**a**) Influence of *D*_it_ on the forward *I*–*V* characteristics. (**b**) Influence of the SRH recombination mechanism on the forward *I*–*V* characteristics.

Figure 7a shows the effect of *D*_it_ on the *I*–*V* characteristics. In this analysis, *D*_it_ was varied from the ideal case of zero to 4.3 × 10^11^ cm^−2^ eV^−1^. The chosen *D*_it_ range covers both the ideal case and the ultralow *D*_it_ regime reported for advanced *β*-Ga_2_O_3_ MIS structures, including values around 8 × 10^10^ cm^−2^ eV^−1^ identified by deep-level transient spectroscopy [53]. It is found that the simulated *I*–*V* characteristics are essentially insensitive to *D*_it_ within this range. This result indicates that the high-quality *h*-BN interface effectively suppresses the impact of interface traps on carrier transport, even when the trap density approaches relatively high levels.

Based on this observation, the bulk SRH recombination mechanism was further examined. Figure 7b compares the *I*–*V* characteristics simulated with and without the SRH recombination model. The nearly perfect overlap of the two curves confirms that, in the vertical Au/*h*-BN/n-type *β*-Ga_2_O_3_ structure, the forward current is dominated by F-N tunneling, whereas the SRH recombination current is negligible. Physically, under positive bias, electrons accumulate at the interface on the n-type *β*-Ga_2_O_3_ side. However, because *β*-Ga_2_O_3_ is an ultra-wide-bandgap semiconductor with an extremely low hole concentration, efficient electron-hole recombination is unlikely. According to SRH recombination theory described in Equation (1), the severe scarcity of minority carriers drives the SRH recombination rate toward zero, effectively eliminating this current path. This substantial suppression of SRH recombination is highly beneficial for optoelectronic applications, as trap-assisted generation–recombination acts as a primary source of dark current that degrades photodetector detectivity [54,55].

**Figure 8:**
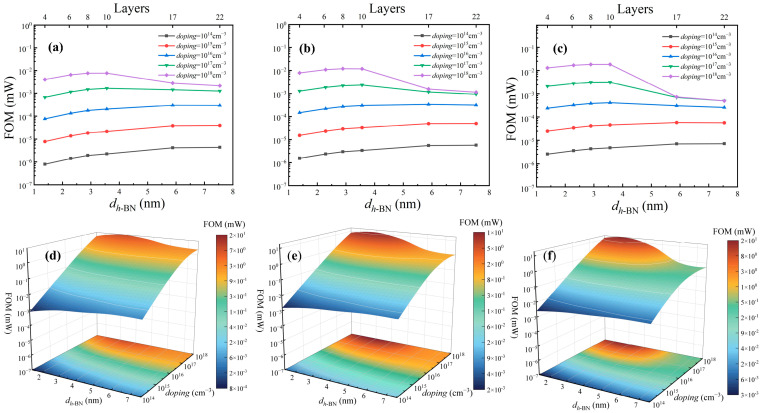
Dependence of the FOM of the MIS structure on metal work function, *h*-BN thickness, and doping concentration. (**a**–**c**) FOM characteristics as functions of *h*-BN thickness for doping concentrations ranging from 10^14^ to 10^18^ cm^−3^ at metal work functions of 4.6, 5.1, and 5.6 eV, respectively. (**d**–**f**) Corresponding three-dimensional FOM surfaces as functions of *h*-BN thickness and doping concentration at metal work functions of 4.6, 5.1, and 5.6 eV, respectively.

Figure 8 illustrates the evolution of the FOM as a function of the semiconductor drift-region doping concentration and the *h*-BN tunneling-layer thickness (*d*_h-BN_) under different WF (4.6, 5.1, and 5.6 eV). In the high-doping regime, the FOM exhibits a clear nonmonotonic dependence on *d_h_*_-BN_, which is attributed to the different sensitivities of *V*_th_ and *R*_on_ to the insulator thickness. Although increasing the doping concentration generally enhances the baseline FOM by increasing the free-carrier density, it also causes the peak of the FOM curve to shift systematically toward smaller *d_h_*_-BN_ values. This shift occurs because heavy doping significantly reduces the intrinsic bulk resistance of the semiconductor layer, thereby making the F–N tunneling resistance of the *h*-BN insulator the dominant component of the total series resistance. Consequently, at high doping levels, even a slight increase in the tunneling-layer thickness leads to a substantial increase in *R*_on_, causing the FOM turning point to appear at a smaller *d_h_*_-BN_. In addition, the metal work function further determines the optimal operating window by modifying the effective interfacial barrier. As the WF increases from 4.6 eV to 5.6 eV, the increased barrier height strengthens the suppression of electron injection and further aggravates the degradation of *R*_on_. To compensate for the forward-conduction degradation caused by the higher energy barrier, the tunneling barrier thickness must be minimized. This drives the optimal *h*-BN thickness further downward, eventually approaching *d_h_*_-BN_ = 3.56 nm for the highest WF. Overall, these results indicate that the theoretical maximum FOM can be achieved through a synergistic design that pairs a high WF metal contact with an ultrathin van der Waals tunneling dielectric. This design mitigates the forward-conduction penalty arising from the combined effects of the high energy barrier and the physical barrier.

**Figure 9:**
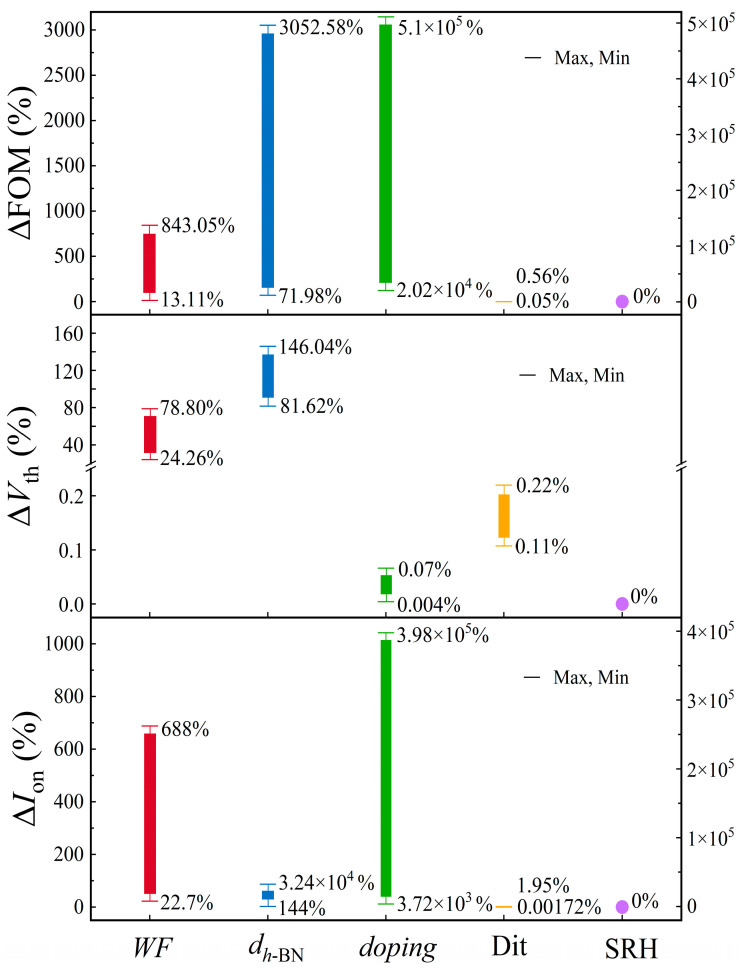
Relative variation ranges of FOM, *V*_th_, and *I*_on_ of the Ga_2_O_3_-based MIS structure in response to different physical parameters.

To quantitatively evaluate the impact of various physical variables on the macroscopic electrical characteristics, a comprehensive sensitivity analysis is presented in Figure 9. The relative variations (ΔFOM, Δ*V*_th_, and Δ*I*_on_) clearly identify the metal WF, *d_h_*_-BN_, and doping as the three dominant performance-dictating parameters. Specifically, *V*_th_ is strongly modulated by the synergistic effect of *d_h_*_-BN_ and WF—which jointly define the composite tunneling barrier, but remains virtually immune to doping variations (<0.1%). Conversely, *I*_on_ and the overall FOM exhibit profound sensitivity to the doping concentration and *d_h_*_-BN_, reflecting their respective roles in modulating the intrinsic bulk resistance and the interfacial tunneling probability. Within the investigated parameter range, non-ideal factors, including *D*_it_ and SRH recombination, exert only a limited influence on the overall device performance, with a maximum variation of less than 2%. This comparatively weak sensitivity suggests that, under the interface conditions considered in this work, the atomically flat *h*-BN interlayer can effectively suppress interface-induced degradation. As a result, the device characteristics are governed primarily by controllable transport parameters rather than by interface-related non-idealities. Taken together, these results demonstrate the potential of a high quality 2D van der Waals interface to mitigate the detrimental effects of interface traps and recombination.

**Figure 10:**
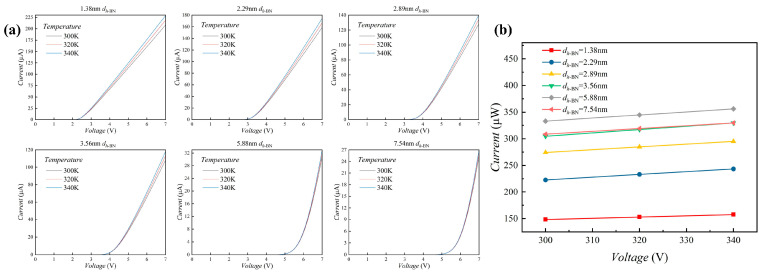
Temperature-dependent electrical characteristics of the metal/*h*-BN/*β*-Ga_2_O_3_ MIS structures. (**a**) Forward *I*–*V* characteristics at 300, 320, and 340 K for different *h*-BN thicknesses. (**b**) Forward current extracted at 7 V as a function of temperature.

Temperature-dependent simulations were performed from 300 to 340 K for different *h*-BN thicknesses. As shown in Figure 10a,b, the forward current increases moderately with temperature, while the overall shapes of the *I–V* curves remain nearly unchanged. More importantly, all investigated *h*-BN thicknesses exhibit similar temperature-dependent trends, and their relative performance differences are preserved. These results indicate that the predicted thickness-dependent optimization remains stable within the investigated temperature range.

**Figure 11:**
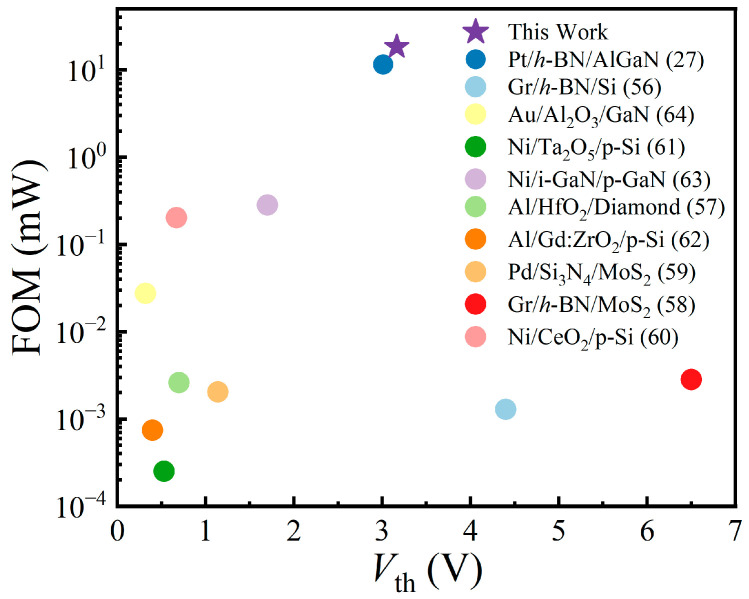
Benchmark of maximum FOM versus *V*_th_ of this work comparing the proposed device with previously reported MIS literature. The optimized *h*-BN/*β*-Ga_2_O_3_ architecture exhibits a significant theoretical advantage, achieving a superior peak FOM in the 10^1^ mW range alongside a *V*_th_ of 3.1 V.

To rigorously benchmark the efficacy of this optimization framework, Figure 11 compares the peak figure of merit of the proposed architecture against recently reported MIS and heterojunction devices [27,56,57,58,59,60,61,62,63,64]. The optimized *h*-BN/*β*-Ga_2_O_3_ device exhibits a formidable theoretical advantage, achieving a peak FOM in the 10^1^ mW range. This metric outperforms existing Si-, GaN-, and transition metal dichalcogenide (TMDC)-based counterparts by one to four orders of magnitude. Prior experimental demonstrations have generally involved a tradeoff between threshold voltage and conduction efficiency. Achieving a high *V*_th_ often incurs a substantial increase in *R*_on_, as exemplified by the Gr/*h*-BN/MoS_2_ device with *V*_th_ > 6 V and an FOM of 10^−3^ mW, whereas maintaining efficient conduction typically limits *V*_th_ to below 1 V. In contrast, the proposed device achieves a higher FOM while maintaining a theoretically predicted *V*th of approximately 3.1 V. Ultimately, these results theoretically indicate that the interface engineering of *h*-BN/*β*-Ga_2_O_3_ is a promising strategy to address conventional performance bottlenecks in advanced heterojunction devices.

## 4. Conclusions

This work systematically investigates the physical mechanisms and performance optimization of a van der Waals MIS heterostructure. Through calibrated simulations of baseline MS junctions, we first demonstrated that structurally imperfect interfaces, particularly the Au/*β*-Ga_2_O_3_ contact, are highly susceptible to severe defect states that trigger anomalous trap-assisted leakage. Such defect-mediated leakage represents a universal bottleneck, limiting power device efficiency and driving dark current generation in emerging *β*-Ga_2_O_3_ optoelectronics. To theoretically address this interfacial degradation, an atomically flat 2D *h*-BN interlayer was simulated to construct a high-quality van der Waals heterointerface. Comprehensive energy-band analysis elucidates that this insertion fundamentally reshapes the conventional single Schottky contact into a composite tunneling barrier system. Specifically, the introduced high potential barrier and improved interface effectively suppress conventional thermionic emission and defect-mediated leakage pathways. Furthermore, because the applied forward bias predominantly drops across the ultrathin dielectric, the severely tilted insulator band forms a triangular barrier, thereby shifting the dominant conduction mechanism to high-field F-N tunneling.

Building upon this foundation, a comprehensive multidimensional engineering strategy was employed to mitigate the inherent physical tradeoff between *V*th and *R*_on_. By systematically scaling *d_h_*_-BN_, WF, and drift-region doping, an optimal structural window was identified. These results indicate the theoretical potential of 2D *h*-BN integration to improve the performance balance compared to conventional devices, establishing critical theoretical guidelines for the design of next-generation, high-performance ultra-wide bandgap power electronics and advanced photodetectors.

## Figures and Tables

**Table 2:** Parameters used in the F–N tunneling mode.

*h*-BN Layers (N)	*A*_e_ (×10^−6^)	*B*_e_ (×10^8^)	*h*-BN Effective Mass (*m*_0_)
4	2.105	1.169	0.366
6	2.112	1.167	0.365
8	2.114	1.166	0.364
10	2.471	1.079	0.312
17	2.734	1.026	0.282
22	4.183	0.829	0.184

## Data Availability

The original contributions presented in this study are included in the article. Further inquiries can be directed at the corresponding authors.

## Abbreviations

The following abbreviations are used in this manuscript:

F–N	Fowler–Nordheim
2D	Two-dimensional
MS	Metal/semiconductor
MIS	Metal/insulator/semiconductor
MIM	Metal/insulator/metal
BFOM	The Baliga figure of merit
TAT	Trap assist tunneling
SRH	Shockley–Read–Hall
*D*_it_	The interface trap density
*V*th	The threshold voltage
*I*_on_	The on-state current
$R_{net}^{SRH}$	The SRH recombination rate
*E*_trap_	The energy offset between the intrinsic Fermi level and the trap level
*d_h_*_-BN_	The thickness of *h*-BN
*I*–*V*	The current–voltage
*R_on_*	The on-state resistance
*F*_ins_	The magnitudes of the electric field in h-BN
WF	Work function
FOM	The power figure-of-merit
